# Detection of Variants in 15 Genes in 87 Unrelated Chinese Patients with Leber Congenital Amaurosis

**DOI:** 10.1371/journal.pone.0019458

**Published:** 2011-05-13

**Authors:** Lin Li, Xueshan Xiao, Shiqiang Li, Xiaoyun Jia, Panfeng Wang, Xiangming Guo, Xiaodong Jiao, Qingjiong Zhang, J. Fielding Hejtmancik

**Affiliations:** 1 State Key Laboratory of Ophthalmology, Zhongshan Ophthalmic Center, Sun Yat-Sen University, Guangzhou, Guangdong, China; 2 Ophthalmic Genetics and Visual Function Branch, National Eye Institute, National Institutes of Health, Bethesda, Maryland, United States of America; Ohio State University Medical Center, United States of America

## Abstract

**Background:**

Leber congenital amaurosis (LCA) is the earliest onset and most severe form of hereditary retinal dystrophy. So far, full spectrum of variations in the 15 genes known to cause LCA has not been systemically evaluated in East Asians. Therefore, we performed comprehensive detection of variants in these 15 genes in 87 unrelated Han Chinese patients with LCA.

**Methodology/Principal Findings:**

The 51 most frequently mutated exons and introns in the 15 genes were selected for an initial scan using cycle sequencing. All the remaining exons in 11 of the 15 genes were subsequently sequenced. Fifty-three different variants were identified in 44 of the 87 patients (50.6%), involving 78 of the 88 alleles (11 homozygous and 56 heterozygous variants). Of the 53 variants, 35 (66%) were novel pathogenic mutations. In these Chinese patients, variants in *GUCY2D* are the most common cause of LCA (16.1% cases), followed by *CRB1* (11.5%), *RPGRIP1* (8%), *RPE65* (5.7%), *SPATA7* (4.6%), *CEP290* (4.6%), *CRX* (3.4%), *LCA5* (2.3%), *MERTK* (2.3%), *AIPL1* (1.1%), and *RDH12* (1.1%). This differs from the variation spectrum described in other populations. An initial scan of 55 of 215 PCR amplicons, including 214 exons and 1 intron, detected 83.3% (65/78) of the mutant alleles ultimately found in these 87 patients. In addition, sequencing only 9 exons would detect over 50% of the identified variants and require less than 5% of the labor and cost of comprehensive sequencing for all exons.

**Conclusions/Significance:**

Our results suggest that specific difference in the variation spectrum found in LCA patients from the Han Chinese and other populations are related by ethnicity. Sequencing exons in order of decreasing risk is a cost-effective way to identify causative mutations responsible for LCA, especially in the context of genetic counseling for individual patients in a clinical setting.

## Introduction

Leber congenital amaurosis (LCA, MIM 204000) is a severe form of inherited retinal dystrophy, characterized by severe visual loss at or near birth, Franceschetti's oculo-digital sign, searching or wandering nystagmus, and pigmentary retinopathy [1], [2]. Visual acuity is rarely better than 20/400 [3]. Fundus changes are extremely variable, ranging from a normal appearance to an obvious pigmentary retinopathy similar to retinitis pigmentosa. Electroretinogram (ERG) recordings are usually extinguished or severely subnormal. In most cases, LCA shows an autosomal recessive pattern of inheritance. However, several families with autosomal dominant LCA have been well documented [2]. The prevalence of LCA is around 1–2 per 80,000 live births, accounting for approximately 20% of cases of inherited blindness among children and more than 5% of all congenital retinopathies [4]. Currently, 16 loci for LCA have been mapped, at which mutations in 15 genes have been identified as being responsible for the disease: *GUCY2D* [5], *CRB1* [6], *RPE65* [7], *RPGRIP1* [8], *AIPL1* [9], *LCA5* [10], *CRX* [11], *LRAT* [12], *TULP1* [13], *RDH12* [14], *CEP290* [15], *RD3* [16], *SPATA7* [17], *IMPDH1* [18], and *MERTK* [19].

It has been estimated that mutations in these 15 genes are responsible for about 65% of all LCA cases [2]. The most frequently mutated genes in published studies are *CEP290* (15%), followed by *GUCY2D* (12%), *CRB1* (10%), *RPE65* (6%), *AIPL1* (5.3%), *RPGRIP1* (4.2%), *LCA5* (1.8%), *CRX* (1.0%), and *MERTK* (0.6%). However, the full frequency spectrum of variation in these 15 genes has not been evaluated in East Asia, an area containing one third of the world’s population. In this study, we performed a comprehensive evaluation of variation in these 15 genes in 87 unrelated Han Chinese patients with LCA.

## Methods

### LCA Patient cohort

Eighty-seven unrelated patients with LCA were recruited at the Pediatric and Genetic Clinic in the Eye Hospital of Zhongshan Ophthalmic Center from 1996 to 2008. They were of Han Chinese ethnicity and lived in southern China. Of the 87 patients, 75 were isolated cases, 9 were from families with autosomal recessive LCA, and 3 were from families with autosomal dominant LCA. Genomic DNA from each patient was prepared from leukocytes of peripheral venous blood by whole blood lysis, followed by phenol-chloroform extraction and ethanol precipitation. The DNA pellet was dissolved in TE buffer (pH 8.0). Genomic DNA was also obtained from 96 unrelated healthy Han Chinese individuals with normal corrected visual acuity and no symptoms or family history of retinal degeneration.

### Ethics

Written informed consent was obtained from participants before the study, conforming to the tenets of the Declaration of Helsinki and following the Guidance for Sample Collection of Human Genetic Disease (National 863-Plan) by the Ministry of Public Health of China. This study was approved by the Institute Review Board of the Zhongshan Ophthalmic Center and the National Eye Institute.

### Mutation screening

The most frequently mutated exons in the 15 genes, plus one intron, were selected based on previous reports for the initial scan for variation in the 87 patients [2]. The primer pairs (Table S1) used to amplify genomic fragments encompassing each of the exons and the adjacent 100 bp or more flanking intronic regions were designed using Primer 3 (http://frodo.wi.mit.edu/primer3/). PCR amplifications were carried out in 10-µl reactions containing 40 ng genomic DNA. Touchdown PCR amplification consisted of a denaturizing step at 95°C for 5 minutes, followed by 35 cycles of amplification (at 95°C for 30 seconds, at 64∼57°C for 30 seconds starting from 64°C with decreasing by 0.5°C every cycle for 14 cycles until remaining at 57°C for 21 cycles, and at 72°C for 40 seconds), and a final extension at 72°C for 10 minutes. The amplicons were sequenced with an ABI BigDye Terminator cycle sequencing kit v3.1 (Applied Biosystems, Foster City, CA), electrophoresed on an ABI3100 or ABI 3130 Genetic Analyzer (Applied Biosystems, Foster City, CA), and analyzed with Seqman software (Lasergene 8.0, DNASTAR, Madison, WI) or Mutation Surveyor (SoftGenetics, State College, PA).

Subsequently, all the remaining exons in 11 of the 15 genes (*GUCY2D*, *CRB1*, *RPE65*, *RPGRIP1*, *AIPL1*, *LCA5*, *CRX*, *SPATA7*, *CEP290*, *RDH12* and *MERTK*) were amplified and sequenced for all patients. For the remaining 4 genes including *TULP1*, *RD3*, *LRAT*, and *IMPDH1*, because of the rarity of reported variation in these genes and exons, only exons with previously reported variants were analyzed. The exons analyzed in our initial scan and subsequent analyses are listed in Table 1.

**Table 1:** The exons and one intron sequenced in our initial screen and in subsequent analyses.

Gene	Total number of exons	Exons for initial scan	Exons for subsequent sequencing
*GUCY2D*	19	2, 3, 8–12,15–17	All remaining
*CRB1*	12	2,6,7,9,11	All remaining
*RPGRIP1*	24	2–4,12,15,16, 21–23	All remaining
*SPATA7*	12	8,11,12	All remaining
*RPE65*	14	3,4,9,10	All remaining
*CRX*	3	3	All remaining
*LCA5*	7	1,2,7	All remaining
*AIPL1*	6	2,5,6	All remaining
*RDH12*	7	5	All remaining
*CEP290*	54	IVS26+1665, 36, 41	All remaining
*MERTK*	19	4,7,14,15,19	All remaining
*IMPDH1*	17	6–8	None
*LRAT*	3	2	None
*RD3*	3	2	None
*TULP1*	15	12–14	None

A variant was predicted to be pathogenic if it was predicted to be damaging by Blosum 62, predicted to be possibly damaging (a value of 1.5 or greater) by the position specific independent counts algorithm of Polyphen [20], had a SIFT score (which distinguishes tolerated variants from those that are not tolerated) equal to or less than 0.05, and was absent in 192 ethnically matched control chromosomes. Splicing changes were predicted by Automated Splice Site Analyses (https://splice.uwo.ca/).

## Results

Fifty-three different variants were identified in 44 of the 87 patients (50.6%), involving 78 of the 88 alleles (11 homozygous and 56 heterozygous) (Tables S2 and S3). These include 8 homozygous or 12 compound heterozygous cases, 5 cases in whom two mutations in one gene and a single mutation in a second gene were identified (triallelic), 3 patients in whom two mutations in different genes were identified (digenic), and 16 patients in whom only a single heterozygous variant was identified. Of the 53 variants identified, 35 (66%) were novel and predicted to be pathogenic, 9 (17%) were known to be pathogenic, and 9 (17%) were novel neutral or unknown effects. An initial scan of 55 amplicons (out of 215 in total, 25.6%) detected 83.3% (65/78) of the mutant alleles detected by the full scan. Sequencing the remaining exons in 11 of the 15 genes detected only 13 additional mutant alleles (16.9%).

The percentage of patients who had variants in the 15 genes were, in decreasing order: *GUCY2D* 16.1% (14/87), *CRB1* 11.5% (10/87), *RPGRIP1* 8% (7/87), *RPE65* 5.7% (5/87), *SPATA7* 4.6% (4/87), *CEP290* 4.6% (4/87), *CRX* 3.4% (3/87), *LCA5* 2.3% (2/87), *MERTK 2.3%* (2/87), *AIPL1* 1.1% (1/87), and *RDH12* 1.1% (1/87) (all variants were taking into account for those patients who had variants in more than one gene). For individual genes, the majority of variants were found in one exon (*RPE65*, *CRX*, *LCA5*, *AIPL1*, and *RDH12*), two exons (*CRB1*, *RPGRIP1*, *SPATA7*, *CEP290* and *MERTK*), or three exons (*GUCY2D*) (Table 2). Further analysis revealed that 9 exons (4.19% fragments sequenced, 9/215) contained 50.8% (39/78) of the variant alleles detected in this study (Table S4). Polymorphisms detected in these genes are shown in Table S5. Clinical data for the 44 patients in which variants were found were listed in Table S6.

**Table 2:** Frequencies of variant alleles detected in the individual exons of each gene.

Gene	Exons in decreasing order (%)								
*GUCY2D*	Exon 2 (30.4)	Exon 11 (13.1)	Exon 12 (13.1)	Exon 3 (8.7)	Exon 4 (8.7)	Exon 8 (8.7)	Exon 9 (8.7)	Exon 16 (4.3)	Exon 17 (4.3)
*CRB1*	Exon 6 (35.3)	Exon11 (29.4)	Exon 9 (23.5)	Exon 7 (5.9)	Exon 4 (5.9)				
*RPGRIP1*	Exon 3 (36.3)	Exon 4 (18.2)	Exon 5 (9.1)	Exon 12 (9.1)	Exon 15 (9.1)	Exon 21 (9.1)	Exon 23 (9.1)		
*SPATA7*	Exon 2 (40)	Exon 11 (40)	Exon 8 (20)						
*CEP290*	Exon 6 (50)	Exon 37 (16.7)	Exon 42 (16.7)	Exon 49 (16.6)					
*RPE65*	Exon 4 (66.6)	Exon 9 (16.7)	Exon 10 (16.7)						
*CRX*	Exon 3 (66.7)	Exon 2 (33.3)							
*LCA5*	Exon 7 (50)	Exon 2 (50)							
*AIPL1*	Exon 6 (100)								
*MERTK*	Exon 4 (50)	Exon 19 (50)							
*RDH12*	Exon 3 (100)								

### *GUCY2D*

Fifteen *GUCY2D* variants (including13 novel variants) were identified in 14 LCA patients (Tables S2 and S3). Variants were found in the homozygous, compound heterozygous, and heterozygous states in 4, 5, and 5 patients, respectively. Of these 14 patients, 6 also had variants in other genes, carrying 2, 3, 4 variant alleles overall. The c.164C>T (p.T55M) variant was found in four patients (one homozygote and three heterozygotes), suggesting an existence of mutation hot spot at this site, since each of those patients have different local SNP haplotypes (data not shown). The c.935C>T (p.T312M) and c.2302C>T (p.R768W) mutations have been described previously. The most frequently mutated 10 exons contained 86.9% (20/23) of the mutant alleles detected by sequencing all 19 coding exons. Variants were most frequent in exon 2 (7 variants found in 6 patients), followed by exons 11 and 12. Besides, several polymorphisms were detected, including c.61T>C (p.W21R), c.154G>T (p.A52S), and c.2101C>T (p.P701S), respectively (Table S5). The c.61T>C (p.W21R) was reported as a polymorphism [21] even though PolyPhen predicted it to be possibly damaging. No variants were found in exons 1, 5–7, 10, 13–15, 18, or 19 (Table 2).

### *CRB1*

Nine variants (7 novel variants) were detected in 10 patients (Tables S2 and S3), including 5 missense, 3 splicing, and 1 nonsense variant(s). The c.866C>T (p.T289M) variant in patient LH7 was reported as a pathogenic mutation based on a study of Italian patients [22], although it was predicted to be benign by Polyphen and tolerated by SIFT. However, it was found not to co-segregate with the disease in another study [23], suggesting it has a nonpathogenic role. The heterozygous variant c.4005+2T>G in LH29 was predicted to abolish the splicing site, with Automated Splice Site Analysis predicting that binding energy would be decreased to 0, and on that basis is predicted to be responsible for disease. The heterozygous 1903T>C (p.S635P) variant in LH29 might be a neutral based on the prediction of Polyphen and SIFT. Patient QT453 and LH16 carried putative triallelic variants (Tables S2 and S3). In our initial scan, sequencing 5 out of the 12 exons detected 94.1% of the mutant alleles (16/17) (Tables 1 and 2). A novel missense variation, c.664G>A (p.E222K), was considered to be an undiscovered polymorphism due to its presence in 3 normal controls. Variants were most often located in exons 6, 9, and 11 (Table 2) but were absent in exon 1, 3, 5, 10, and 12.

### *RPGRIP1*

Eight novel *RPGRIP1* variants were identified in 7 patients, including 7 pathogenic mutations and 1 putatively neutral variant. Of the 7 patients, 3 had a single heterozygous variant and 3 were compound heterozygotes. The other patient, RP208, had four variants in three genes: a homozygous c.535delG (p.E179SfsX10) variant in *RPGRIP1*, a heterozygous c.1312C>T (p.R438C) variant in *GUCY2D*, and a heterozygous c.295G>A (p.V99I) variant in RPE65. The novel c. 3388G>C (p.E1130Q) variant in individual QT654 was predicted to be neutral by both PolyPhen and SIFT. Most of these variants occurred within the RPGR interacting domain. The efficiency of the initial screening was 90.1 % (10/11 alleles) (Tables 1 and 2). In addition, three known polymorphisms were detected in LCA patients (Table S5). Variants were most frequent in exon 3 (Table 2), and no variation was found in exons 1, 2, 6, 8–11, 16–20, 22, and 24.

### *RPE65*

Three variants were detected in 5 patients, including one known (c.1059_1060insG/p.K354EfsX11) [24] and two novel variants (c.295G>A/p.V99I, c.997G>C/p.G333R) (Table S2). The two novel variants were predicted to be neutral by PolyPhen and SIFT. Four patients carried the same heterozygous c.295G>A (p.V99I) variant that is absent in 96 normal controls. One patient was a compound heterozygote for two mutations (c.997G>C and c.1059_1060insG). Our initial scan of 4 of the 14 exons detected 100% of the variant alleles (6/6) identified after sequencing all 14 exons.

### *SPATA7*

Three variants in 4 patients were found in *SPATA7,* 2 were novel and 1 known (Tables S2 and S3). Two variants were predicted to be pathogenic and the c.995T>C (p.I332T) variant was predicted to be neutral by Polyphen and SIFT. The variants were heterozygous in three patients and homozygous in one patient. Patient LH15 was heterozygous for a known 4 bp deletion in *SPATA7* and a common missense mutation in *GUCY2D* (p.T55M). Our initial scan on 3 of the 12 exons could find 60% of the mutant alleles (3/5) detected after all 12 exons were sequenced.

### *CEP290*

First, we analyzed the genomic regions encompassing IVS26+1655, exon 36, and exon 41 of *CEP290*, where the c.2991+1655A>G is the most common mutation in Caucasians. However, no variation was detected in the 87 Chinese patients, except for a presumably neutral variant, c.5709+25A>C.

Five variants in the other CEP290 exons were subsequently detected in 4 patients, including 4 that were novel (c.367C>T, c.4897C>T, c. 6766delC, and c.6787A>G) and 1 that was previously described (c.383_386delATAG). Two patients were compound heterozygotes and another two were single heterozygotes. The effect of the c.6787A>G (p.S2263G) variant could not be predicted by PolyPhen but SIFT labeled it as damaging (Tables S2 and S3).

### *CRX*

Three heterozygous variants in *CRX* were identified in 3 patients (Tables S2 and S3). All 3 variations are predicted to be pathogenic. Of the 3, the c.541delG (p. A181PfsX5) variant in patient LH9 and the c.458delC(p.P153QfsX34) variant in RP178 were reported by our previously study [25], [26].

### *LCA5*

Two novel heterozygous variants in *LCA5* were identified in two patients, respectively (Tables S2 and S3). The 1820_1821delCA (p.Q607VfsX6) variant in exon 7 is predicted to cause a frameshift and the c.634G>T (p.A212S) variant in exon 2 is predicted to be benign by both PolyPhen and SIFT. Additionally, two known polymorphisms, p.D26A and p.G656D, were found in 35 and 32 patients, respectively (Table S5).

### *AIPL1*

A novel homozygous variant, c.926_927insCCTGAACCGCAGGGAGCT (p. E309DinsLNRREL), was identified in patient QT338 (Tables S2 and S3). In addition, a known polymorphism, c.268G>C (p.D90H), was detected in 31 patients (Table S5).

### *RDH12*

One novel heterozygous c.236C>T (p.A79V) variant was detected in patient LH16, and was predicted to be benign (Tables S2 and S3). In addition, p.R161Q, a known polymorphism, was detected in 3 patients (Table S5).

### *MERTK*

Two novel heterozygous variants in *MERTK* were identified in patients LH28 and RP143. Both of them are predicted to be pathogenic. Sequencing revealed that patient RP143 was heterozygous at two sites: c.2873C>T (p.P958L) in *MERTK* and c.6787A>G (p.S2263G) in *CEP290* (Tables S2 and S3).

### *TULP1*, *RD3*, *LRAT* and *IMPDH1*

Unlike the other 11 genes listed above, for which all coding exons were analyzed by sequencing, only those exons with previously reported variants were analyzed in *TULP1*, *RD3*, *LRAT*, and *IMPDH1*, due to the rarity of reported variants in the exons of these genes. No variants were detected in any of the exons screened in these genes, including exons 12–14 of *TULP1*, exon 2 of *RD3*, exon 2 of *LRAT*, and exons 6–8 of *IMPDH1*.

## Discussion

Leber congenital amaurosis is the earliest occurring and most severe inherited retinal dystrophy. Since the initial identification of mutations to *GUCY2D* as a cause of LCA in 1996 [5], mutations in a total of 15 genes have been identified as being responsible for LCA, accounting for approximately 65%–70% of LCA cases [2], [27], [28]. However, the variant frequencies for these genes vary between different ethnic groups. In northern Pakistan, the genes most commonly mutated in LCA are *RPGRIP1* (29% of families), *AIPL1* (21% of families), and *LCA5* (21% of families); whereas in Caucasian populations, mutations in *RPGRIP1*, *AIPL1*, and *LCA5* account for only 4.2%, 5.3%, and 1.8% of LCA cases, respectively [2]. Although *CEP290* is the most commonly identified mutant gene in Caucasian LCA patients, accounting for 15% of LCA cases [29], mutations in *CEP290* have not been detected in LCA patients from Korea [30], Saudi Arabia [31], northern Pakistan [32], or southern India [33]. An understanding of the frequency spectrum of variants in these 15 genes in different populations will not only facilitate genetic diagnosis and genetic counseling for this disastrous disorder, but will also identify patients who may potentially benefit from gene therapy or other possible interventions [31]. Because the *NPHP5* gene was only recently reported as related to LCA by Stone EM, et al [34], this gene was not included into our study.

Here, we performed comprehensive mutational analysis of 15 genes in 87 unrelated Chinese patients with LCA. Of the 15 genes, all coding exons in 11 genes were analyzed by cycle sequencing. For the remaining 4 genes which have only infrequently been identified as causal for LCA, only exons in which variants had previously been identified were sequenced. The percentage of patients who had variants in the 15 genes were, in decreasing order: *GUCY2D* 16.1% (14/87), *CRB1* 11.5% (10/87), *RPGRIP1* 8% (7/87), *RPE65* 5.7% (5/87), *SPATA7* 4.6% (4/87), *CEP290* 4.6% (4/87), *CRX* 3.4% (3/87), *LCA5* 2.3% (2/87), *MERTK 2.3%* (2/87), *AIPL1* 1.1% (1/87), and *RDH12* 1.1% (1/87). Thus, the frequencies of variants in different genes vary remarkably in different populations. *GUCY2D* was the gene most frequently mutated in Chinese patients while the genes found to be most frequently mutated in other populations were *RPGRIP1* in people from northern Pakistan [32], *CRB1* in the Spanish [28], and *CEP290* in Caucasians [15]. The overall rate variant detection was 50.6% (44/87) in this study, which is comparable to the worldwide variations detection rate of 65% if the difference between the frequency of variants in *CEP290* is taken into consideration (*CEP290* mutations occur in 15% of Caucasian patients but in only in 6.4% of Chinese patients) [2].

Homozygous mutations were identified in a number of cases, often related to the family and population structure. For example, family LH22 was having homozygous T55M mutations in *GUCY2D*, families LH32 and QT585 showing homozygous S611P mutations in *CRB1*, and family RP208 showing a single base deletion in *RPGRIP1* are from consanguineous matings in an isolated Chaoshan population. Families QT521 and QT608 showing splicing and M784R mutations in *GUCY2D* respectively, family LH24 with a R395X mutation in *SPATA7* and family QT338 showing an 18 base pair insertion in *AIPL1* are not know to be consanguineous, but are from isolated populations.

Identifying the most frequently mutated exons in these 15 genes will greatly facilitate detection of variation in LCA patients in a clinical testing. Our initial scan of 25.6% (55 of 215) of exons, based on the frequencies of variation in other populations, detected 83.3% (65/78) of all the mutant alleles we detected. This indicates that reducing the amount of labor and cost by 75% would still result in detection of over three quarters of the variation in our samples. Based on our study of 87 Chinese patients, sequencing only the 9 most polymorphic (4.18% of the 215 sequenced regions) exons (exons 2, 11, and 12 of *GUCY2D*; exons 6, 11, and 9 of *CRB1*; exon 4 of *RPE65*; exon 3 of *RPGRIP1*; *and* exon 6 of *CEP290*) would result in detection of 50.8% (39/78) of all variants found in this study. This would use less than 5% of the labor and cost of a comprehensive sequencing strategy and detect over 50% of the variation present in other groups of Chinese patients. Screening these exons in order of amount of expected variation would be predicted to cut expenses significantly while still being accurate and comprehensive. Our results provide a key bridge between bench and bed side and should make genetic diagnose of LCA in Chinese patients more accessible and practical. This should greatly enhance the clinical genetic counseling, diagnosis, and early intervention of LCA in the Chinese population. These results also highlight the importance of analyzing the causative genes and their exons in different ethnic groups in a systematic and population-specific fashion.

Another important point from our study is that some sequence variations might mistakenly be thought to be causative mutations for LCA if only a single individual gene were analyzed. For example, the heterozygous c.164C>T (p.T55M) variant in *GUCY2D* might be incorrectly labeled a common mutation for LCA because it was found in 3 unrelated patients with LCA but not in 96 controls, was predicted to be a pathogenic mutation by Polyphen, and was identified as a causative mutation in SIFT. This might be the case for patient LH22, who had homozygous c.164C>T mutations, but it may not cause LCA in patient QT453 since QT453 had homozygous nonsense mutations (G1226X) in *CRB1*. Even though both mutations themselves could be pathogenic, digenic mutations may not necessarily cause disease, since one of the phenotypically normal parents in families LH16, QT479, QT453, QT509, and RP208 presumably carry digenic mutations. In this study, digenic variants were detected in 3 patients, including LH15 (*GUCY2D* and *SPATA7*), QT659 (*CRB1* and *LCA5*), and RP143 (*CEP290* and *MERTK*). Whether these mutations are indeed causative and what types of digenic variations may be responsible for LCA needs additional study. For the 5 patients (LH16, QT479, QT509, QT453, and RP208) with 3 or 4 variants in two or 3 genes, their clinical phenotypes are comparable to patients with homozygous or compound heterozygous mutations in a single gene (Table S6), suggesting that the third or fourth mutant allele may not have an additive effect and may be more likely to act as a benign variant. In such cases, the 6 additional variant alleles in these 5 patients should not be counted toward the total number of mutant alleles and, therefore, the adjusted number of mutant alleles detected should be 72, not 78 (from 44 patients). In an earlier genotyping microarray study, Zernant, et al. determined 7.3% of LCA patients carry a third mutant allele [35], a greater fraction than would be expected by chance. On the other hand, single heterozygous variants were detected in genes known to cause autosomal recessive LCA in 16 patients (15 predicted to be pathogenic mutations and 1 predicted to be neutral). This might simply represent a failure to detect a second variant. Conversely, it is possible that neutral or silent variants or polymorphisms in the 15 genes sequenced here or in other genes might serve as disease modifier alleles with another major gene defect occurring simultaneously [36]. Additional comprehensive studies, or perhaps further work with animal models, are necessary to answer this question.

In conclusion, systematic analysis of the full frequency spectrum of variation in the 15 selected genes not only gives us an overview of the molecular etiology of LCA in Chinese but also provides useful biomarkers for genetic counseling. In the near future, patients in whom pathogenic mutations are identified could become potential participants for gene therapy, and thus, identification of efficient and effective diagnostic approaches based on the population genetics specific to the patient will become increasingly important.

## Supporting Information

Table S1 **Primers used for sequencing.** This table listed 340 primers used to amplify genomic fragments of the *GUCY2D*, *CRB1*, *RPE65*, *RPGRIP1*, *AIPL1*, *SPATA7*, *LCA5*, *CRX*, *RDH12*, *CEP290*, *IMPDH1*, *TULP1*, and *MERTK* genes.
(XLS)

Table S2 **Variants detected in the 15 genes sequenced in 87 unrelated LCA patients and 96 healthy controls.** Mutations in each gene were listed separately. a) The value is the difference between the original value and substitution value. b) These changes are likely neutral variations based on computational prediction. c) Controversial, as some reports regard this mutation as a non-pathogenic mutation, den Hollander et al 2004.
(XLS)

Table S3 **Variants detected in the 15 genes sequenced in 87 unrelated LCA patients and 96 healthy controls.** Patients with muations were separated into three groups. Group A: Patients with homozygous or compound heterozygous mutations in one gene. Group B: Patients with only one heterozygous mutation in one gene. Group C: Patients with Digenic or Triallelic or 4 variants. a) The difference between the original value and the substitution value. b) These variants are likely neutral variations based on computational prediction. c) Controversial, as some reports regard this mutation as an non-pathogenic mutation, den Hollander et al 2004.
(XLS)

Table S4 **Frequencies of the 78 variant alleles in individual exons.** The frequency of mutations in each exon was calculated based on either all mutant alleles in all 15 genes or those in individual gene.
(XLS)

Table S5 **Polymorphisms detected in 87 unrelated LCA patients and 96 healthy controls.** ND: not determined. a) The c.2101C>T (p.P701S) variant in *GUCY2D* has been reported to cosegregate with LCA in three pedigrees (Zernant et al., 2005). This variant was found at similar frequencies in the patients and the normal controls in our study (28 heterozygotes and 1 homozygotes out of 87 LCA patients and 26 heterozygotes and 2 homozygote in 96 normal controls). The high frequency of this variant, and especially its occurrence in a heterozygous state in normal controls, argues strongly against its classification as a mutation (Vallespin et al., 2007).
(XLS)

Table S6 **Clinical data for the 44 patients who carry variants in the 15 sequenced genes.** FMB = First few months after birth *Cons = consanguinity marriage of parents. ©The boy and his father are both affected. # Both of the twins are affected. PV = poor vision; PP = photophobia; NB: Night blindness; NYS = nystagmus; ODS = oculodigital sign; RN = roving nystagmus; AV = attenuated vesseles; CRD = carpet-like retinal degeneration; PIG = pigment deposit; NFR = no foveal reflex; MA: Macular atrophy.
(XLS)
